# OSAS: its burden increases, not enough the awareness

**DOI:** 10.1186/s40248-018-0156-1

**Published:** 2018-12-03

**Authors:** Antonio Sanna, Donato Lacedonia

**Affiliations:** 1 Chairman of “Sleep-related Breathing Disorders Working Group” – Italian Respiratory Society (SIP/IRS); Pneumology Unit, San Jacopo Hospital, Pistoia, Italy; 2 Department of Medical and Surgical Sciences, Institute of Respiratory Diseases, University of Foggia, Foggia, Italy

Obstructive sleep apnoea syndrome (OSAS) is a chronic disease that is widespread in the world and affects subjects of both sexes at all ages. The prevalence of moderate and severe OSAS is extraordinarily high, with values of 23.4% in women and 49.7% in men over 40 years old [1]. These values are similar to those of other chronic diseases like arterial hypertension (up to 43% in women and 48% in men) [2], or higher like diabetes (9.6% in women and 10.3% in men) [3]. OSAS is associated with many comorbidities (stroke, cardiovascular, metabolic and neurocognitive disease, cancer) [4, 5], and motor vehicle [6] and work accidents [7]. For these reasons, OSAS has a significant adverse impact on quality of life [8] and life expectancy [9].

There is a very significant gap between the estimated number of patients with OSAS (approximately 80% of them are undiagnosed) and the ability of health systems to diagnose and care patients [10]. This gap is likely to increase further for three main reasons: (i) the increase in the prevalence of OSAS is associated with increased obesity, with obesity being the highest risk factor for OSAS [11]; (ii) the prevalence of OSAS increases with age, with the life expectancy being increasing at least in economically advanced countries [11]; and (iii) to prevent motor vehicle accidents and related injuries, protocols or mandatory testing for the screening and early diagnosis of OSAS in commercial and/or private vehicle drivers have been either proposed and/or adopted [12]. The burden and economic impact of OSAS on social and health systems are already very high and difficult to manage; they will be increasingly more in the future.

For many years, it has been known and accepted that the continuous positive airway pressure (CPAP) is the treatment of choice for OSAS, regardless of severity. Indeed, the CPAP treatment is associated with an improvement in quality of life and life expectancy by preventing the occurrence of comorbidities or improving their control. It also reduces the number of traffic and work accidents [5]. In the last few years, the approach to OSAS has changed. Different OSAS phenotypes have been defined [13] and it has been shown that, at least in mild and moderate OSAS, the oral appliance and new techniques for upper airway surgery offer therapeutic success and outcomes similar to those of CPAP. Personalised medicine can, therefore, also be applied in the management of OSAS patients.

In order to counteract the impact of OSAS on public health, in 2016, the Italian Minister of Health has planned and approved a new approach that is aimed at improving the health of OSAS patients [10]. This is based on the involvement of any dentist or physician, including general practitioners and paediatricians in any outpatient clinic, to formulate a clinical suspicion of OSAS by using a structured interview. It is expected that this new and holistic approach increases early diagnosis and will allow easy access to diagnosis and treatment for an increasing number of patients with suspected OSAS. It will also be possible by using the help provided by new technologies and telemedicine [14]. Despite the awareness of OSAS as a health issue of great relevance increasing, as shown by statements on the early diagnosis or prevention of traffic and work accidents approved by health administrations and agencies, a significant number of individuals with OSAS are unaware of their condition. Furthermore, a very high number of physicians are unaware of the consequences of OSAS and that OSAS can be cured with an improvement of the individual quality of life as well of the social welfare.

The Sleep-Related Breathing Disorders Working Group of the Italian Respiratory Society (IRS) wanted to publish the thematic series “Obstructive Sleep Apnea Syndrome: an emerging chronic disease” with the aim to help all physicians, not only the pneumonologists, to improve their awareness of OSAS and provide them with up-to-date knowledge on the prevention, diagnosis, consequences, therapy and management of OSAS today and in the immediate future.

With the invaluable contribution of some of the leading international experts in this field (see respectively the scheduled papers by Cistulli et al., Bonsignore MR et al., Marin JM et al.), it was possible to write and publish this thematic series for *Multidisciplinary Respiratory Medicine.* The Series aims to be an in-depth analysis on OSAS, considering comorbidities, treatment approaches, limitations and innovative therapies for individual tailoring and future perspectives of this common sleep related respiratory disorder.
